# Factors Affecting the Bioproduction of Resveratrol by Grapevine Cell Cultures under Elicitation

**DOI:** 10.3390/biom13101529

**Published:** 2023-10-16

**Authors:** Juan Carlos Vera-Urbina, Susana Sellés-Marchart, Ascensión Martínez-Márquez, María José Martínez-Esteso, María Angeles Pedreño, Jaime Morante-Carriel, Roque Bru-Martínez

**Affiliations:** 1 Departamento Bioquímica y Biología Molecular y Edafología y Química Agrícola, Facultad de Ciencias, Universidad de Alicante, 03690 Alicante, Spain; jcvera75@gmail.com (J.C.V.-U.); susana.selles@ua.es (S.S.-M.); asun.martinez@ua.es (A.M.-M.); mjose.martinez@ua.es (M.J.M.-E.); jaime.morante@ua.es (J.M.-C.); 2 Department of Plant Biology, Faculty of Biology, Campus de Espinardo, University of Murcia, 30100 Murcia, Spain; mpedreno@um.es; 3 Department of Plant Biotechnology, Faculty of Forestry and Agricultural Sciences, Quevedo State Technical University, Quevedo 120503, Ecuador; 4 Instituto de Investigación Sanitaria y Biomédica de Alicante ISABIAL-Fundación Para el Fomento de la Investigación Sanitaria y Biomédica de la Comunitat Valenciana FISABIO, 03010 Alicante, Spain

**Keywords:** *Vitis vinifera*, stilbene, resveratrol, bioreactor, plant cell culture, bioproduction, methyl jasmonate, methyl-β-cyclodextrin

## Abstract

Here we present a study of the characterization and optimization of the production of trans-Resveratrol (*t*-R) in grape (*Vitis vinifera* cv. Gamay) cell cultures elicited with methyl jasmonate (MeJA) and dimethyl-β-cyclodextrin (DIMEB). The aim of this study was to determine the influence of a number of factors of the grapevine cell culture on *t*-R production level in 250 mL shaken flasks that would enable the better control of this bioproduction system when it is upscaled to a 2 L stirred bioreactor. The factors included the optimal growth phase for elicitation, the concentration of elicitors and of biomass, the order of addition of elicitors, and the illumination regime and ageing of cells. We found out that the optimal biomass density for the production of *t*-R was 19% (*w*/*v*) with an optimal ratio of 0.5 g DIMEB/g biomass. The most productive concentrations of the elicitors tested were 50 mM DIMEB and 100 µM MeJA, reaching maximum values of 4.18 mg·mL^−1^ and 16.3 mg·g biomass^−1^ of *t*-R concentration and specific production, respectively. We found that the order of elicitor addition matters since, as compared with the simultaneous addition of both elicitors, the addition of MeJA 48 h before DIMEB results in ca. 40% less *t*-R production, whilst there is no significant difference when MeJA is added 48 h after DIMEB. Upon upscaling, the better conditions tested for *t*-R production were aeration at 1.7 vol/vol/min without agitation, 24 °C, and 30 g·L^−1^ sucrose, achieving production rates similar to those obtained in shaken flasks.

## 1. Introduction

Resveratrol trans-isomer (*t*-R) is a natural stilbene that acts as a phytoalexin in several plant species, including grapevine [1]. In biomedicine, this antioxidant compound is well known for its multiple pharmacological properties such as cardioprotective, antitumor, and neuroprotective activities [2], as well as its antifungal and antibacterial properties [3]. Also, it has been shown that *t*-R increases lifespan in several model organisms [4]. These health-beneficial properties and the natural origin of this multifunctional molecule have also attracted the nutraceutical and cosmetic sectors, and currently, there is an increasing demand for *t*-R. Bioproduction is one of the most promising sustainable strategies of *t*-R procurement, which includes metabolically engineered microorganisms and wild plant cell systems from which grapevine cell suspensions have the advantage of high productivity, scalability, and safety [5]. 

The biosynthesis of *t*-R and its derivatives, such as the glycosylated form piceid, in grapevine tissues can be induced by biotic and abiotic elicitors [6,7]. In grapevine cell cultures, elicitor-induced *t*-R biosynthesis leads to the advantageous extracellular accumulation of this compound [8,9,10,11,12], which is particularly abundant, in the range of g/L, when cells are elicited with modified cyclodextrins such as dimethyl-β-cyclodextrin (DIMEB) either alone [13,14,15] or combined with the phytohormone methyl jasmonate (MeJA) [16,17,18,19,20,21] or its structural analog coronatine [22]. Jasmonic acid and its volatile ester (MeJA), synthesized via the octadecanoid pathway [23], are generally considered to be inducers of the expression of genes for the biosynthesis of secondary metabolites [17,24,25] and for proteins related to pathogenesis (PR) [10,17,26,27]. In grapevine cell suspensions, MeJA alone caused a four-fold increase in the intracellular accumulation of piceid, but not of *t*-R [28], while extracellular production was in the range of a few mg/L [9]. Elicitations performed on Monastrell grapevine cell suspensions with the combination of DIMEB and MeJA showed a synergistic effect since the extracellular accumulation of *t*-R was much greater than in the sum of the individual elicitations; the observed effect of MeJA slowing down cell division combined with a strong elicitor such as DIMEB could be responsible for the high production of *t*-R, which was consistent with the expression of the genes of the stilbenoid pathway [16]. An omics analysis of an elicited grapevine cell culture revealed an induction level of genes from shikimate, phenylpropanoid, and stilbenoid pathways, besides MYB transcription factors, that agreed with the synergistic effect of the combined elicitation of DIMEB and MeJA [18,21]. The successful combination of elicitor-modified cyclodextrins and MeJA for the production of *t*-R in cell suspensions has been imitated for the production of valuable phytochemicals from different families, including phenylpropanoids, terpenes, alkaloids, and quinones [29].

Production scale-up has also been addressed in bioreactors of different types and working volumes stimulating the culture, with elicitors such as chitosan [30], MeJA [31], β-cyclodextrin and MeJA [32], and DIMEB alone or combined with MeJA [33,34,35], with the aim of enhancing the production of stilbenes and promoting extracellular accumulation for commercial application.

From the above studies, it can be assessed that modified cyclodextrins such as DIMEB are a key elicitor for a stable extracellular accumulation and that MeJA combined with DIMEB causes a synergistic effect multiplying several-fold the amount of *t*-R and other phytochemicals accumulated in the extracellular medium. However, little is known about how different factors and variables of the cell culture relevant to the implementation of a production process influence the yield of *t*-R. Thus, the goal of this piece of research is the systematic study of the influence of a number of factors of the grapevine cell culture on *t*-R production level in shaken flasks, to enable better control of the grapevine cell culture as a *t*-R bioproduction system. The factors include the optimal growth phase for elicitation, the concentration of elicitors and of biomass, the order of addition of elicitors, and the illumination regime and aging of cells. Moreover, other factors specific to the upscaling of the culture such as the influence of aeration-agitation, temperature, and carbon source concentration were studied in a laboratory commercial bioreactor.

## 2. Materials and Methods

### 2.1. Plant Material

A *V. vinifera* L. cv. Gamay callus was kindly supplied by Drs. J. C. Pech and A. Latché (ENSA, Toulouse, France) in 1989. The callus was maintained by monthly subculturing onto fresh standard medium as described elsewhere [14], consisting of 3.1 g/L Gamborg B5 medium with 2% (*w*/*v*) sucrose as a carbon source, growth regulators (0.2 mg·L^−1^ kinetin, 0.1 mg·L^−1^ 1-Naphthaleneacetic acid), 0.250 g·L^−1^ casein hydrolysate, Morel vitamins [36], and 8 g·L^−1^ agar as a solidifying agent. To initiate cell suspensions, the callus was transferred into 200 mL of fresh standard medium without agar in a 500 mL Erlenmeyer flask and shaken at 110 rpm in a rotary shaker incubator, and then it was maintained by subculturing every 14 days when the stationary phase was reached, under the same conditions as above. Both the callus and cell suspension were grown under 6 W·m^−2^ of light intensity, with a photoperiod of 16 h in light and 8 h in darkness at 24 °C.

### 2.2. Elicitor Treatment

Elicitation was carried out as described in [14] with slight modifications. Briefly, a weighted amount of filtered and washed cells was transferred into a 250 mL Erlenmeyer flask and suspended in sterile fresh standard medium without agar that contained elicitors at a final volume of 100 mL. Elicitors were either doubly methylated-β-cyclodextrin at hydroxyls 2 and 6 (DIMEB; CAVASOL^®^ W7M, Merck-Sigma, Darmstadt, Germany) or methyl jasmonate (MeJA), or both together. The cell suspension was incubated with continuous rotary shaking (100 rpm) at 25 °C and during the photoperiod as described above, unless otherwise stated. After 96 h of incubation (unless otherwise stated), cells were harvested by filtration under a slight vacuum, and the spent medium was used for the analysis of *t*-R. Each data point was recorded in triplicate.

For elicitation in the Biostat B 2 L bioreactor (BBraun Biotech Intl GmbH, Melsungen, Germany), the jar containing 1 L of the medium supplemented with 50 mM DIMEB together with the calibrated pH electrode was previously sterilized. Then, the dissolved oxygen probe was calibrated after cooling down to the operating temperature, following manufacturer instructions. Then, 250 g of cells drained under a gentle vacuum were transferred into the bioreactor jar, and the process was run in different conditions of aeration-agitation, temperature, and sucrose concentration, as detailed in Table S1, and at constant concentrations of elicitor and biomass.

### 2.3. Determination of Stilbenoids

One hundred microliters of the cell-free medium obtained by gentle vacuum filtration of the cell culture was properly diluted with water and methanol to a final methanol concentration of 80% (*v*/*v*). For stilbenoid determination, 30 μL of sample passed through an Anopore 0.2 μm filter was analyzed by liquid chromatography in an Agilent 1100 series HPLC equipped with UV–vis detector as described elsewhere [16]. The sample injected into a LiChrospher 100 RP-18 column (250 × 4 mm, particle size 5 μm, column temperature 35 °C) was eluted in a gradient of solvents A (0.05% TFA) and B (0.05% TFA in methanol:acetonitrile 60:40 *v*/*v*), at a flow rate of 1 mL/min. The gradient consisted of: 0 min. 10% B; 5 min, 15% B; 40 min, 35% B; 45 min, 65% B; 50 min, 65% B; 55 min 10% B. The trans-Resveratrol (*t*-R) and trans-Piceid (*t*-P) standards were obtained from ChromaDex Inc. (Irvine, CA, USA), and under the chromatographic conditions used, their retention times in minutes were: *t*-P 23.5 and *t*-R 31.5 (Figure S1). Calibration curves were constructed for the quantification of the standard compounds in the samples obtained from the cell cultures.

### 2.4. Effect of Growth Phase

In order to study the effect of the growth phase of Gamay cell suspensions on *t*-R production, elicitation with DIMEB was carried out on cells up to 14 days of growth from the subculture, always using a constant biomass density of 19% (*m*/*v*) and harvesting after 96 h of incubation. The assays were performed in triplicates in 125 mL Erlenmeyer flasks with 50 mL of culture.

### 2.5. Effect of DIMEB Concentration and Cell Density

To study the effect of the concentration of DIMEB on the production of *t*-R, elicitations were performed in 100 mL flasks with a constant cell density of 12% (*m*/*v*) and incubated for 96 h. For the effect of cell density, the amount of DIMEB was kept constant at 50 mM. Various amounts of fresh biomass from washed and filtered cells were weighed into 500 mL Erlenmeyer flasks and elicitation medium with 50 mM DIMEB was added to a volume of 250 mL to achieve cell concentrations between 3 and 45% (*m*/*v*). To study the combination of both factors, approximately 4, 13, 24, 30, 45, and 56 g of fresh cell biomass were weighed into 500 mL volumetric Erlenmeyer flasks followed by the addition of 70 mL of Gamborg B5 medium to each flask containing either 2, 6.5, 12.0, 15, 22.5 or 28 g DIMEB so that a constant ratio of 0.5 g/g biomass could be achieved. Finally, the 125 mL operating volume was completed with Gamborg B5 medium to obtain cell densities of 3, 10, 19, 24, 36, and 45% (*m*/*v*), respectively. Each assay was conducted in triplicates.

### 2.6. Elicitor Mix Concentration Optimization

This experiment uses a 3^2^ factorial design (“Number of levels ^Number of factors^” or 3 × 3) with two quantitative factors—A: concentration of the DIMEB elicitor, and B: concentration of the MeJA elicitor. Three concentration levels—low, intermediate, and high—were considered for each factor. For factor A: DIMEB (15, 35, and 50 mM) and for factor B: MeJA (25, 50 and 100 µM). Nine possible combinations were obtained, and three replicates of each trial were performed. To carry out the experiment, 25 g of fresh biomass was weighed in 250 mL Erlenmeyer flasks and the sterile elicitation medium was added with its corresponding combination to obtain a cell density of 20% (*m*/*v*). The elicitation time was 7 days (168 h).

### 2.7. Effect of Order of Addition of Elicitors

In the case of the addition of MeJA in the first place, “MeJA-pretreated” cell suspensions were held for 48 h in fresh medium containing 100 µM MeJA, whilst “non-pretreated” were held only with fresh medium. After 48 h, the “MeJA-pretreated” cells were supplemented with fresh medium containing concentrated DIMEB and 100 µM MeJA; one-half of the “non-treated” cells were supplemented with fresh medium containing concentrated DIMEB (elicited without MeJA), and the other half was supplemented with both concentrated DIMEB and MeJA (elicited simultaneously with both elicitors). In the case of the addition of MeJA after DIMEB, the group “elicited simultaneously with both elicitors” was prepared by adding the final volume of fresh medium containing 50 mM DIMEB and 100 µM MeJA, and the larger group of “elicited without MeJA” was prepared by adding the final volume of fresh medium containing 50 mM DIMEB. After 48 h, half of the “elicited without MeJA” group was spiked with concentrated MeJA in ethanol to a final concentration of 100 µM, and the other half was spiked only with ethanol. In this way, the final volume and concentration of biomass were constant: the concentration of DIMEB was 50 mM, and the concentration of MeJA, when present, was 100 µM, both before and after the 48 h preincubation period. In both experiments, aliquots were taken under aseptic conditions approximately every 24 h for 7 days to determine the kinetics of *t*-R production. At all times during the experiments, flasks were incubated in a rotary shaker at 110 rpm during the photoperiod.

### 2.8. Effect of Darkness and Ageing

The darkness regime was applied to cultures of two different ages from the moment the callus was dispersed in a liquid medium: Age I of 21 cycles and Age II of 6 cycles of subculturing during the photoperiod. At each age, one set of flasks was maintained and elicited according to points 2.1 and 2.2 in darkness, and as a control, another set of flasks was maintained and elicited during the photoperiod. In each cycle during the experiment, half of the flasks were used for subculturing and the other half for elicitation in fixed conditions of elicitor and biomass concentrations.

A statistical analysis of significance contrast was performed using the Student’s *t*-test for paired data of the average values of the variables’ concentration and the specific production of extracellular *t*-R in the photoperiod and darkness. The null hypothesis for the influence of darkness is: “The darkness regime does NOT influence the production of *t*-R when Gamay grapevine cell suspensions are elicited in dark conditions against the photoperiod.” The null hypothesis for the influence of cell suspension age is: “The age of the culture does NOT influence the production of *t*-R when Gamay grapevine cell suspensions are elicited in dark and photoperiod conditions.” The null hypotheses are accepted if the calculated absolute value of *t* (|*t*|) is lower than the critical value *t* within a 95% confidence interval, taking into account eight trials (cycles) of each experiment. Thus, the critical value for *t* (0.05; 7) is 2.36 (See Tables S3 and S4).

### 2.9. Statistical Analysis

SigmaPlot for Windows version 8.0 software was used for the statistical analysis and graphic representation. To analyze the results of the optimization of *t*-R with two factors (DIMEB and MeJA) and three-level concentrations, a 3^2^ factorial design was used; therefore, nine experiments with three biological replicates were carried out. The concentration of *t*-R was obtained from the average of three analytical replicates from each biological replicate. A response surface design was applied, and the data were adjusted to a polynomial second-order model ($y=\beta_{0}+\beta_{1}x_{1}+\beta_{2}x_{2}+\beta_{12}x_{1}x_{2}+\beta_{11}x_{1}^{2}+\beta 22+x_{2}^{2}+\in$) [37]. This equation was defined in the software to depict a three-dimensional response surface plot of the expected yield of *t*-R as a function of the concentration of DIMEB and MeJA. The ANOVA of the fitted equation shows an R square of 92% (Table S2). For the statistical analysis of darkness and the age effect for *t*-R production in Gamay grapevine cell suspensions, a paired *t*-test was carried out with the SigmaPlot ver 8.0 software.

## 3. Results

### 3.1. Effect of the Cellular Physiological State (Growth Phase) on the Production of t-R by Elicitation with DIMEB

**Figure 1:**
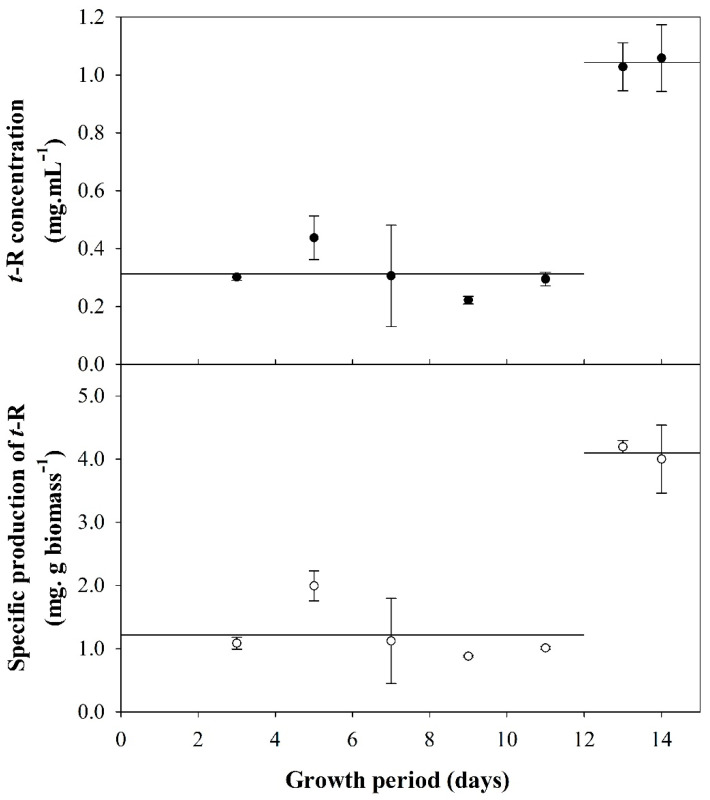
Production of *t*-R elicitation with DIMEB (50 mM) using 19% (*m*/*v*) biomass inoculate at different times of cell growth. Concentration of *t*-R (●) and specific production of *t*-R (○). Data representative of two repeated experiments.

Figure 1 shows that between days 3 and 11, covering the lag and exponential growth phases, a low accumulation of *t*-R, and thus specific production, is obtained, for which the average values are 0.31 ± 0.08 mg·L^−1^ and 1.22 ± 0.44 mg·g biomass^−1^, respectively. On the other hand, the yield of *t*-R achieved on days 13 and 14, covering the stationary phase, increased 3.3-fold with respect to the yield obtained in the previous growth phases. These results demonstrate that the stage of growth has a critical influence on *t*-R yield and that the culture must enter the stationary phase to maximize the yield.

### 3.2. Effect of DIMEB Concentration and Cell Density on t-R Production by Elicitation with DIMEB

**Figure 2:**
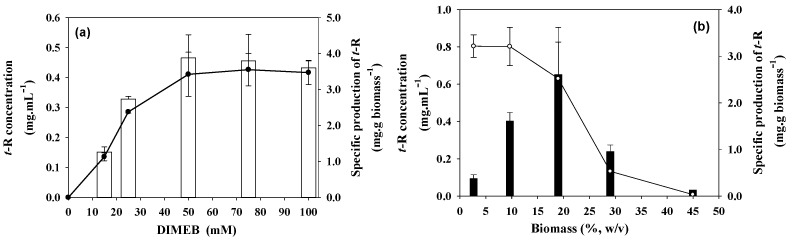
Effect of elicitor concentration and cell density on *t*-R production by grapevine cells after 96 h elicitation. (**a**) Effect of different concentrations of DIMEB on grapevine cell suspensions (*Vitis vinifera* L. cv. Gamay) at 12% density. *t*-R concentration (empty bars) and specific production (-●-). (**b**) Effect of cell density of grapevine cell suspensions (*Vitis vinifera* L. cv. Gamay) on the production of *t*-R by elicitation with 50 mM DIMEB. *t*-R concentration in mg·mL^−1^ (filled bars) and specific production in mg·g biomass^−1^ (-○-). Data representative of two repeated experiments.

Figure 2a shows the results of concentration and specific production of *t*-R in the extracellular medium obtained at different DIMEB concentrations and fixed 12% *w*/*v* cell biomass. As can be seen, the accumulated concentration of *t*-R in the extracellular medium shows an increase proportional to the concentration of DIMEB in the range of 15 to 50 mM, reaching a maximum concentration of 0.46 ± 0.07 mg·mL^−1^, and from there up to 100 mM, production stabilizes.

The effect of the density of the culture was studied at 50 mM DIMEB which was the minimal DIMEB concentration that gave maximal specific production (Figure 2a). Figure 2b shows that the concentration of *t*-R increases sharply with the density of the culture until it reaches a maximum at 19% density, and then it also sharply decreases, with the production at 45% density being less than 10% of that at 19% density. On the other hand, the specific production follows a decreasing sigmoid dependence with the cell density, sharply dropping between 19 and 28% and being very low at the highest density of 45%. The highest value of 0.65 ± 0.25 mg·mL^−1^ *t*-R concentration was reached at 19% (*m*/*v*) cell density, while the maximum value for the specific production (3.2 ± 0.3 mg·g biomass^−1^) occurs between 3 and 10% (*m*/*v*).

**Figure 3:**
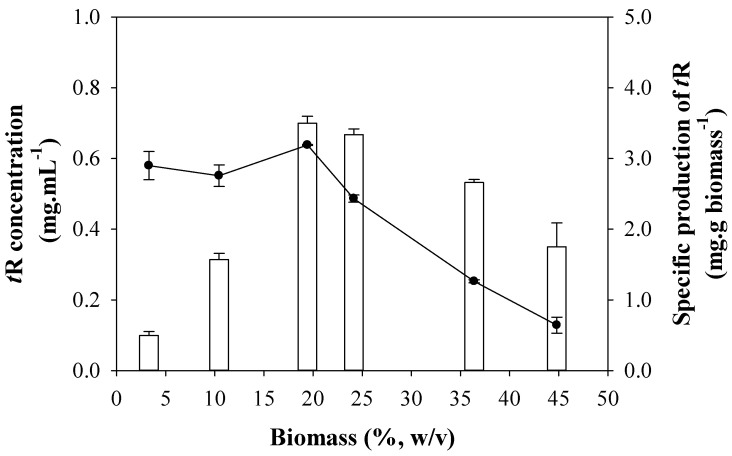
Effect of cell density on the production of *t*-R by elicitation with DIMEB at a fixed ratio of 0.5 g DIMEB/g biomass. *tR* concentration in mg·mL^−1^ (empty bars); *t*-R specific production in mg·g biomass^−1^ (-●-). Data representative of two repeated experiments.

From the combination of both experiments, there seems to be an optimal concentration of elicitor and an optimal concentration of biomass for an efficient production, that gives approximately 0.5 g DIMEB/g biomass. In a new experiment, such a ratio was held constant. Figure 3 shows the results of *t*-R production keeping a fixed DIMEB/biomass ratio. The concentration of *t*-R also increases sharply with the density of the culture between 3 and 19% and then decreases gradually between 19 and 45% biomass. The specific production was constant between 3 and 19%, and then it decreases gradually between 19 and 45% biomass to ca. 30% of the maximal specific production.

As shown in Figure S2, there were important variations in biomass during the 96 h of incubation. This difference was positive for the elicitations carried out with densities of 3 and 10% (*m*/*v*), which increased by 10 and 1%, respectively, while it was negative for the rest, reaching losses of 60%. One factor that may contribute to fresh weight loss is the partial dehydration of cells due to osmotic stress caused by increasing the absolute concentration of DIMEB. According to the results, a 20% dehydration is a stress that can be manageable for the cells and does not affect the performance in the production of *t*-R, but greater losses become harmful to the process. These data, together with observations under the light microscope, confirmed that cell lysis occurs from a certain concentration of DIMEB that harms the production of *t*-R.

### 3.3. Elicitor Mix Concentration Optimization by Factorial Design 3^2^

**Figure 4:**
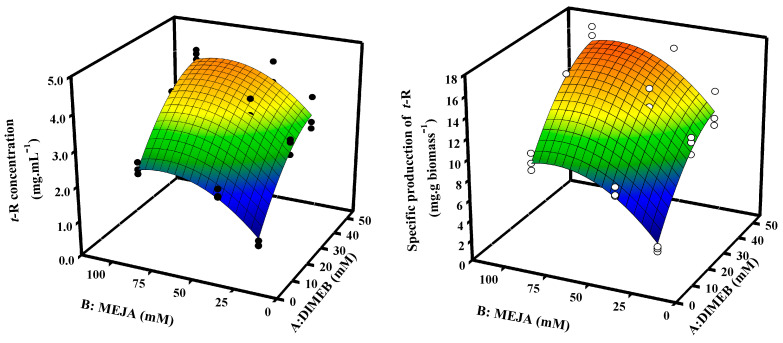
Representation using response surface of factorial design 3^2^ to optimize the production of *t*-R in Gamay grapevine cell suspensions by elicitation with DIMEB and MeJA. Concentration data of *t*-R (●) and specific production of *t*-R (○) both fitted to the response surface analysis regression model.

In order to optimize the concentrations of DIMEB and MeJA in production, a 3^2^ factorial design was applied. Table S2 shows the average concentration and specific production values of *t*-R in the extracellular medium and their analysis of variance (ANOVA). The maximum accumulation is reached with the combination of DIMEB 50 mM and MeJA 100 μM (4.18 ± 0.14 mg·mL^−1^ and 16.3 ± 0.9 mg·g biomass^−1^) and the lowest with the combination of DIMEB 15 mM and MeJA 25 μM (0.57 ± 0.09 mg·mL^−1^ and 1.9 ± 0.3 mg·g biomass^−1^). In order to better visualize the behavior of the data, these were represented as a surface plot (Figure 4), which allowed for adjusting the experimental data to a polynomial of the second degree that describes a data behavior with a smooth hill-like representation. This allows us to obtain the combination of both factors with higher performance for the production of *t*-R—in this case, the combination of the highest levels. The statistical analysis indicates that both the differences found between the levels with respect to factor A (DIMEB) and those found with respect to B (MeJA) are highly significant; however, the increase in *t*-R accumulation produced by the different levels of factor A is independent of the level of factor B, and vice versa, and therefore, there is no significant statistical interaction between factors (Table S2).

The polynomial obtained from said model predicts with 92% confidence the production performance with the desired combination of these variables. The second-degree polynomials obtained from this model are:

For *t*-R concentration

$$y=-2.6878+0.1557A+0.0669B-\left({7.42\times 10}^{-5}\right)A*B-0.0014A^{2}-0.0004B^{2}$$

For *t*-R specific production

$$y=-10.6025+0.5817A+0.2644B-\left({2.0\times 10}^{-4}\right)A*B-0.0053A^{2}-0.0015B^{2}$$

The coefficients and analysis of variance obtained from the response surface model are also presented in Table S2.

### 3.4. Effect of the Order of Addition of Elicitors to Cultures of Gamay Grapevine Cell Suspensions

In the previous experiment, both DIMEB and MeJA elicitors are present when elicitation is initiated, but the order in which cells come into contact with them might have consequences on the final production of *t*-R and shed new light on the cellular response to elicitors. Here, we left a time-lapse of 48 h between the addition of elicitors and compared the extracellular *t*-R production with the addition of DIMEB alone at time zero or added simultaneously with MeJA. MeJA, being a water-insoluble ester, was delivered to the sterile culture medium dissolved in ethanol after sterile filtration. Previous studies have shown that a similar amount of ethanol had no influence on the growth of Monastrell grapevine cell cultures [16].

**Figure 5:**
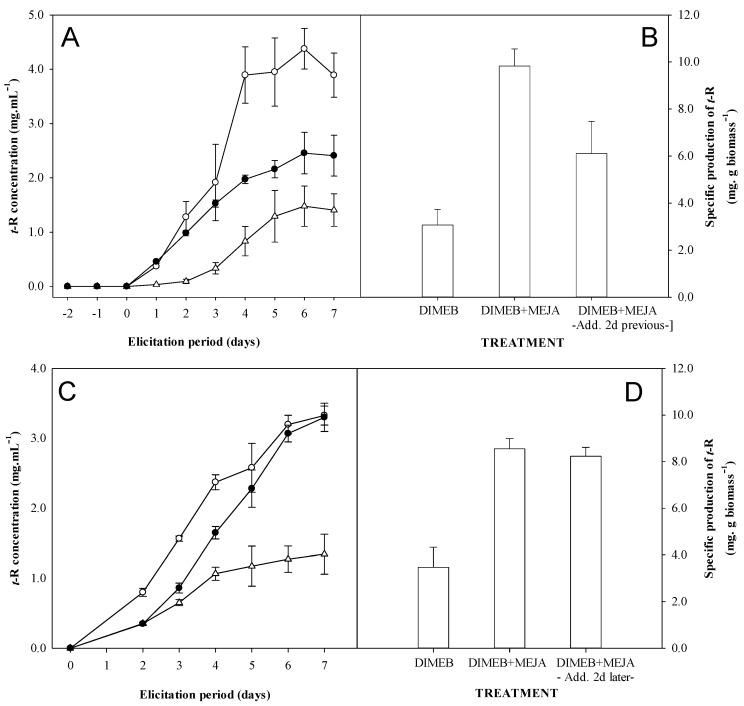
Effect of the addition of the MeJA 2 days prior or after the addition of DIMEB on the production of *t*-R with Gamay grapevine cell suspensions. Charts: (**A**). Kinetics of *t*-R concentration in extracellular medium: DIMEB at *t* = 0 (-∆-), DIMEB + MeJA at *t* = 0 (-○-) and MeJA at *t* = −2, DIMEB at *t* = 0 (-●-). (**B**). Specific production of *t*-R at day 7. (**C**). Kinetics of *t*-R concentration in extracellular medium DIMEB at *t* = 0 (-Δ-), DIMEB + MeJA at *t* = 0 (-○-) and MeJA at *t* = +2, DIMEB at *t* = 0 (-●-). (**D**). Specific production of *t*-R at day 7. Data representative of two repeated experiments.

Figure 5A,B shows the results obtained from the kinetics of extracellular *t*-R production and the specific production at the end of elicitation when MeJA is added first. During the MeJA pre-incubation, no accumulation of *t*-R occurs in the extracellular medium. Only upon the addition of DIMEB *t*-R does accumulation begin in all cases. Both the accumulation rate and the production after 7 days when preincubating with MeJA were found to be intermediate between the control without MeJA and the control with the simultaneous addition of MeJA and DIMEB.

Figure 5C,D shows the results obtained from the kinetics and the specific production at the end of elicitation when MeJA is added after DIMEB. When observing the control treatments of the joint addition of DIMEB and MeJA and DIMEB alone, a 2.3-fold increase in extracellular *t*-R production was observed as early as two days after elicitation. The addition of MeJA at that time causes an acceleration in the accumulation of *t*-R, making it comparable both in kinetics and in final performance to those obtained by adding both components, MeJA and DIMEB, simultaneously.

### 3.5. Effect of Adaptation to the Darkness and Ageing of the Cell Suspension

Table 1 shows the concentration and specific production values of *t*-R obtained from the elicitations carried out in the photoperiod and darkness conditions of cell suspensions with different ages of adaptation in the photoperiod prior to the start of the experiment. For cell suspensions of older age (Age I), the values obtained in the dark show an increase of ≈19% in the accumulation of *t*-R and a ≈25% increase in the specific production of *t*-R; similarly, for cell suspensions of younger age (Age II), dark conditions show a ≈26.5% increase in *t*-R accumulation and a ≈25% increase in specific *t*-R production with respect to the photoperiod. The t statistic (Table S3) rejects the null hypothesis proposed in both ages; therefore, the darkness significantly increases the production of *t*-R (concentration and specific production) during the photoperiod regardless of the age of the culture. Likewise, the t statistic (Table S4) rejects the null hypothesis proposed in both illumination regimes; therefore, the age of the culture significantly influences the production of *t*-R in both darkness and photoperiod conditions.

### 3.6. Factors Affecting t-R Bioproduction in Bioreactor

Metabolite production by plant cell suspensions can be affected by scale factors, such as the passage to a bioreactor from shaken flasks [38]; environmental factors, such as stress caused by hydrodynamic aeration and agitation; and temperature and nutrient availability. We have studied the effect of some factors such as temperature, concentration of carbon source, aeration, and agitation on *t*-R production in a 2 L commercial design stirred tank bioreactor. Table S1 describes the experiment conditions of elicitation and the parameters used.

**Figure 6:**
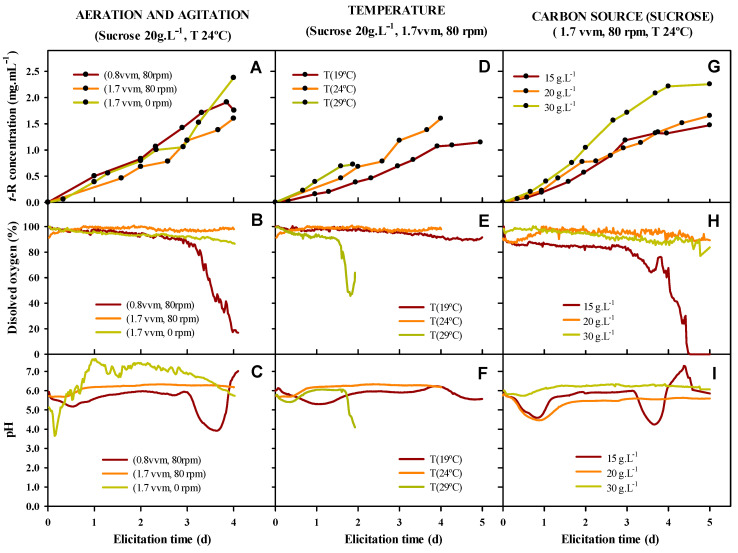
Production of *t*-R (**A**,**D**,**G**) in Biostat B 2 L bioreactor under different conditions of aeration and agitation (**A**–**C**), temperature (**D**–**F**) and carbon source concentration (**G**–**I**). Evolution of dissolved oxygen (**B**,**E**,**H**) and pH (**C**,**F**,**I**) in the culture medium continuously monitored with measuring electrodes.

Figure 6 shows the kinetic of *t*-R production in the extracellular medium, and the evolution of pH and dissolved oxygen in the medium during elicitation.

As can be seen in the middle row of Figure 6 that dissolved oxygen holds practically constant during the elicitation at nearly 100% saturation, except for in some of the tested conditions where a fast drop occurs, indicating a sudden and fast O_2_ consumption likely associated with the stress of the culture. In the bottom row, pH shows a typical drop-and-recovery pattern during the first 24 to 36 h, remaining quite stable for the rest of the elicitation time, but there are some exceptions when a second drop occurs. These exceptions correspond to the condition of low aeration, high temperature, and low concentration of the carbon source. When observing the kinetics of *t*-R accumulation, the dissolved oxygen and pH drop phase is associated with an arrest in the accumulation of *t*-R, suggesting that metabolic stress is occurring and blocks the flow of resources, at least towards this branch of secondary metabolism. Such stress could be of different origins, including insufficient oxygen supply, heat stress, or nutrient depletion. No stress seems to occur at a high aeration rate (1.7 vvm) irrespective of the mechanical agitation, thus highlighting the importance of effective aeration for the fitness of the culture. In fact, in experiments realized only with agitation, the *t*-R production was very low. Likewise, cells appear highly stressed at 29 °C since the dissolved oxygen and pH drop occurs before 48 h, when a culture color change also occurred, turning from dark red to brown as a consequence of cellular lysis and massive cell death. No stress occurred at 19 °C or 24 °C. A decrease in temperature clearly slows down the rate of *t*-R production, as judged from the kinetics during the first 40 h for the three temperatures tested, and during the whole elicitation time for 24 and 19 °C. Carbon source concentration affected both the rate and yield of *t*-R accumulation in a dose-dependent manner. During elicitation, stress (i.e., dissolved oxygen and pH drop) was only observed after three days in 15 g/L sucrose treatment, likely due to carbon source depletion that forced cells to adjust the metabolism and arrest the accumulation of *t*-R.

It is interesting to note that the drop of dissolved oxygen occurred some hours before that of pH; thus, if oxygen consumption is triggered during the elicitation process in a bioreactor equipped with continuous O_2_ monitoring, it could be considered as an indicator of stress and some action could be taken to correct it, such as aeration increase, decrease in temperature, or nutrient feeding.

Table 2 summarizes the results obtained for *t*-R production by Gamay grapevine cell suspensions upon elicitation with DIMEB in the bioreactor under different conditions of temperature, aeration and agitation, and concentration of carbon source. In general, the passage from shaken flasks to the bioreactor leads to enhanced specific production of *t*-R, which is highly desirable. However, the dissolved oxygen and pH traces have revealed that there are operating conditions that produce stress in the culture, and in extreme cases such as high temperature, it may cause massive cell death that also in the degradation of polyphenols by oxidation. Thus, such conditions should be avoided. Summarizing the findings of production, the tested conditions in which the specific and total production were best are high aeration without agitation, 24 °C, and 30 g·L^−1^ sucrose. Because this is an experiment in which some operation conditions were tested for their effect on *t*-R bioproduction, further optimization experiments should be performed to find optimal values of these parameters.

## 4. Discussion

### 4.1. Effect of Factors Related to Cell Physiology, Growth Phase, and Aging, on t-R Bioproduction

Most of the knowledge accumulated on the production of *t*-R and other stilbenoids in grapevine cell cultures is the result of empirical work testing the effect of different elicitors on *t*-R yield and cell culture survival. However, publications to date pay limited attention to the effects of other factors the cell culture has on stilbene production. In this sense, the optimal timing of elicitation is of utmost importance. The physiological state of the cells in culture is determined by the phases of the growth curve (i.e., lag, exponential, and stationary) as well as by the age of the culture in liquid suspension. It has been generally observed that the addition of elicitors between the middle-to-late growth phase results in the best cell culture response in terms of producing secondary metabolites, as compared to previous and later phases [39]. In cell cultures of *Taxus yunnanensis*, using inoculums between 16 to 24 days of growth at various densities with different mixtures of elicitors, the specific production of paclitaxel (taxol) was dependent on the age of the inoculum, obtaining the optimal value with inoculums with 20 days of growth, which corresponds to an early stationary phase [35]. In the case of grapevine cell suspension, the intracellular accumulation of the glycosylated resveratrol derivative piceid upon MeJA elicitation was shown to be higher when added in the exponential phase than in the stationary phase [28]. Likewise, the highest production of saponins by *Panax gingseng* cells treated with yeast elicitor and MeJA was achieved when elicitors were added on the day of inoculation as compared to later days [40]. However, in these experiments, the availability of nutrients in the medium might be also a limiting factor to the production of secondary metabolites as the former are also decreasing as the culture progresses. In *Eschscholzia californica,* it was shown that the production of alkaloids upon chitin elicitation in fresh medium of a weighed amount of cells could be enhanced in cell lines that had been conditioned in P or N nutrient-limiting conditions and that the production level was related to the intracellular accumulation of glucose and phosphate at the time of elicitation [38]. They found that the N-limited cell line was ca. 10-fold more productive than the P-limited one and that the elicitor effect was maximal at the end of the growth phase, when intracellular levels of glucose and phosphate were higher.

Here, we took care that medium composition was the same in all elicitation conditions as well, and only the cells were in different physiological conditions. Then, we could clearly see that when cells reach the stationary phase, they trigger their capacity to produce *t*-R when elicited with DIMEB (Figure 1), not being limited by carbon and energy availability—this agrees very well with the observations in *E. californica* [41]. It might be possible that once nutritional resources are re-supplied to the culture, cells might divide actively again. However, as shown for rose cultures in the stationary phase, most cells that had stopped division and entered a senescent-like status may not divide for quite a long period, even after subculturing in fresh medium [42]. Accordingly, grapevine cells in the stationary phase seem to be prone to use the resources for secondary metabolism rather than growth and division when the cyclodextrin elicitor is present, since, as seen in Figure S2, the biomass undergoes a limited or a negative Increase in fresh weight, suggesting almost no use of resources for growth.

The aging of cell suspensions by long-term subculture may result in the decay of secondary metabolite production. There is experimental evidence that long-term subcultured plant cell lines of medicinal species, such as *Taxus* sp., *Panax* sp., or *Cephalotaxus manii*, among others, reduce their capacity to produce taxanes [43], gingsenosides [44], and cephalotaxine [45], respectively. A six-month-old *Taxus chinensis* cell suspension line accumulated about 12-fold more flavonoids and 4-fold more taxanes than a ten-year-old line [46]. On the other hand, plant cell population diversification in cell cultures due to somaclonal variation may follow different evolutionary models in which, after an initial adjusting phase, the frequency of cells bearing a pursued phenotypic trait may either increase or decrease during long-term maintenance [47]. Here, we have shown that a culture aged after 21 liquid subculture passages, which accounts for ca. 10 months since the callus dispersion, displays a statistically significant 1.4-fold higher *t*-R production capacity than a younger, ca. 3-month-old culture of six subculture passages (Table 1 and Table S3). Given that we have not exerted any specific selective pressure on the liquid culture, it can be assumed that this particular cell line follows a model in which the frequency of highly producing cells increases with age for at least 10 months. In contrast, aged *Vitis amurensis* cell lines have been shown to undergo a decrease in the production of *t*-R, and the treatment with a DNA demethylating agent could enhance the yield by two-fold [48], thus suggesting that DNA methylation could be a mechanism of somaclonal variation involved in the long-term decrease in *t*-R production capacity in these cell lines.

### 4.2. Effect of Factors Related to Elicitation Handling: Biomass Density and Elicitors Concentration, on t-R Bioproduction

The concentration of the elicitor and of the biomass have also a deep influence on specific production. Different studies on the production of *t*-R using grapevine cell cultures elicited with cyclodextrins show rather heterogeneous results [15,19,22] to which differences in biomass and elicitor concentration, elicitor incubation time, and genotype may contribute significantly. Belchi-Navarro et al. [19] observed in grapevine cv Monastrell cell cultures elicited with DIMEB that a four-fold increase in biomass caused a ten-fold decrease in the specific production of *t*-R, and they explained that the cell reaction could rise when the cell quantity is low by increasing the number of receptors for elicitors in cell membranes. Lambert et al. [35] also observed in *Vitis labrusca* cell suspensions elicited with MeJA that the specific production of stilbenes, mainly *t*-R and pallidol, was higher at the lower biomass concentration. The results in Figure 2, which are in agreement with these observations, show that *t*-R specific production has a limit, and the limiting factor can be either the cell biomass (Figure 2b), since production levels off at certain DIMEB concentration, or the concentration of DIMEB (Figure 2a), since the excess of biomass over DIMEB causes a reversion of the elicitor effect, i.e., a strong decrease in specific production. In other words, specific production seems to decrease or increase according to the mass ratio of DIMEB/biomass rather than the elicitor concentration itself. A plausible explanation for this behavior would be the existence of a finite number of DIMEB interaction sites per cell, thus suggesting a specific type of interaction. In *Vitis labrusca,* it was observed that the MeJA concentration-to-biomass ratio is a key element in understanding *t*-R production, and the optimal ratio found in that work was 0.125 mmol/g DW [35]. In Figure 2a, the limit of specific production is achieved at 50 mM DIMEB for a 12% cell density, accounting for ca. 0.54 g DIMEB/g biomass. In Figure 2b, for 50 mM DIMEB, specific production starts decreasing above 10% of cell density, which accounts for ratios below 0.6. According to this hypothesis, the result of *t*-R specific production expected at a fixed DIMEB/biomass ratio (for example, 0.5, as assayed in Figure 3) would be constant and not vary whatever the cell density. This prediction is partially fulfilled—between 3 and 19% (*m*/*v*). At higher cell densities, the production is lower than expected, but even so, it is higher than at equivalent densities at a fixed concentration of elicitor, as seen in Figure 2b. The deviation from the hypothesis above 19% biomass may be due to other factors that become relevant under certain experimental conditions, such as osmotic stress, that affect the physiology of the cell. Thus, during the separation of cells and broth after incubation, elicitor changes in cell color as the concentration of the DIMEB increased were observed, going from red to brown, with the formation of larger aggregates. Likewise, the broth color also changed, gradually turning from amber to brown. The brown hue in the broths was observed at the highest cell and elicitor concentrations and could be a consequence of oxidation processes caused by cell lysis (Figure S3). Therefore, productivity seems to rely on the DIMEB/biomass ratio, limited by a maximum concentration of DIMEB that can cause irreversible damage to the cells. This fact has direct biotechnological implications and should be strictly controlled in *t*-R production processes based on grapevine cell cultures.

As demonstrated in previous studies, DIMEB and MeJA have a synergistic effect on the extracellular accumulation of *t*-R [16]. We can find other examples of the synergistic effect of the combined action of MeJA with other elicitors in the bibliography. MeJA with 2-hydroxypropyl-β-cyclodextrin increased the intracellular production of anthraquinones in suspension cultures of *Rubia tinctorum* [49]. The ginsenoside biosynthesis-related genes and ginsenoside accumulation were highly induced by 100 μM MeJA in combination with 200 μM of sodium nitroprusside in adventitious root cultures of Panax ginseng [50]. A combination of 0.1 mM MeJA and 0.1 mM SA in the immobilized cells of Ginkgo biloba increased the production of bilobalide and ginkgolides A, B, and C more than in the unelicited cultures [51]. Taxol biosynthesis was clearly increased by the joint action of methyl jasmonate and cyclodextrins (CDs), reaching production levels 55 times higher than in non-elicited cultures [52].

Here, we also study the effect of the mixed concentration of both elicitors on *t*-R production. Due to the costs of elicitors, particularly DIMEB and other cyclodextrins, it would be desirable to find optimal elicitor mixes to reduce costs of the process. The conditions tested indicate an optimal concentration of 50 mM DIMEB and 100 µM MeJA (Figure 4). Although the optimal concentrations coincide with the highest values tested, the results presented in Figure 2 as well as the shape of the response surface in Figure 4 suggest that higher concentrations of DIMEB might even be counterproductive. Likewise, studies in Monastrell cell suspensions show that above 100 µM MeJA the *t*-R yield decreased [19]. The equations that fit the response curve can be used to adjust the MeJA concentration at a given cost-limiting DIMEB concentration.

### 4.3. Effect of the Order of Addition of Elicitors on t-R Bioproduction

Also, we found that the order of addition of elicitors matters in the production of *t*-R (Figure 5). The delivery of MeJA 48 h before DIMEB causes a decrease in the *t*-R production compared to the addition of both elicitors at the same time, though it is higher than eliciting only with DIMEB. Conversely, the addition of MeJA 48 h after DIMEB does not cause a significant effect on the final *t*-R production, just a delay in the accumulation curve. This could be interpreted as the cells have “memorized” which compound they first interacted with, which determines the final production of *t*-R when the reference conditions are set, that is, both elicitors are present. The data support the hypothesis that the addition of DIMEB could relieve or avoid a repressive mechanism activated by MeJA that allows *t*-R maximal production levels, comparable to the simultaneous addition of both elicitors. Also, Sabater-Jara et al. [52], who found a synergistic production of taxol due to MeJA and CD elicitation, showed that the addition of MeJA alone to the cell cultures resulted in a reduced growth (20–30%) compared with the control cells, as was reported by other authors in various cell cultures [19,53]. However, the addition of MeJA to *Taxus* cell cultures, which have been previously treated with CDs, reduces this negative effect of MeJA as the cell biomass (g dry weight/L) at the end of the experiment was almost identical to that of the control cells, and cell viability was only slightly affected. Thus, a sort of “memory” seems to be exhibited also in *Taxus* cell cultures.

The JA signaling pathway promotes the proteasomal degradation of JAZ proteins bound to JA-Ile, the bioactive form of JA and MeJA, thus allowing for the transcription of the JAZ-repressed genes [54,55,56]. JA signaling was shown to be involved in the transcriptional activation of the *VvMYB14* transcription factor [57], and on the other hand, it has been shown that VvMYB14 and VvMYB15 bind to *STILBENE SYNTHASE* (*STS*) gene promoters to induce their expression, which relates with the accumulation of stilbenes, especially piceid [58,59]. In grapevine cells, MeJA induced the expression of *VvMYB5B* [21], which is known to regulate the phenylpropanoid pathway in grapevines [60]. Also in grapevine cells, MeJA induced the expression of phenylpropanoid pathway genes and of *STS*, but no extracellular *t*-R was produced [16], as also observed here during the 48 h before the addition of DIMEB (Figure 5A). Moreover, the mixed treatment of DIMEB and MeJA was found to induce *VvMYB15* expression [21] and, although the microarray used did not contain probe sets representing *VvMYB14*, Hurtado-Gaitan et al. showed that *VvMYB14* is also induced by DIMEB and is coordinately accumulated along with transcripts for *VvSTS36* and *VvSTS29* and *t*-R in both Gamay cell cultures and leaves [61]. It was recently shown that VvWRKY8, which expresses upon *t*-R accumulation, binds to VvMYB14, thus avoiding the expression of *STS* genes and closing a regulatory loop, fine-tuning the *t*-R synthesis in grapevines [62]. In this regulatory loop, VvMYB30, whose expression is induced by *t*-R and VvWRKY8, binds to the same elements as VvMYB14 in the *STS* promoter, repressing the expression [63].

It is well established that only the presence of DIMEB enables the extracellular accumulation of *t*-R. Hypothetically, this fact would keep the intracellular levels below the threshold needed for *VvWRKY8* and *VvMYB30* induction (although there is no evidence for this yet), thus explaining the long-lasting biosynthesis and accumulation at high levels outside the cells observed in our experiments (Figure 4) and previous studies [14]. The late steps of this DIMEB-induced pathway include at least the expression of *VvMYB14*, *VvSTSs* and *VvGSTU10*, encoding a GST protein also induced by DIMEB involved in the extracellular accumulation of *t*-R [64]. Taking into account that MeJA induces *STS* genes but does not lead to the extracellular accumulation of *t*-R, nor to the induction of *VvGSTU10*, it is reasonable to speculate that MeJA might also induce the expression of the repressor VvWRKY8 via the *VvMYB14*/*VvSTSs*/*t*-R intracellular production and that such a repressor might remain in the cell after DIMEB addition, reducing the level of *t*-R production. So, it can be hypothesized that grapevine cells might “memorize” their previous contact with MeJA through the accumulation of the negative *t*-R biosynthesis regulators VvWKRY8 and VvMYB30. On the other hand, the addition of MeJA 48 h after DIMEB only accelerates the biosynthesis rate and thus the maximal level of *t*-R can be reached just with some delay, compared with the simultaneous addition of both elicitors (Figure 4). To date, no one positive regulator opposed to the action of VvWRKY8 has been described, but its hypothetical existence would align well with the synergistic effect of combining DIMEB with MeJA and the accelerating effect of adding MeJA 48 after DIMEB. In this sense, Vannozzi et al. [59] have identified a number of novel candidate TFs putatively involved in *STS* regulation belonging to other gene families than *MYBs* by constructing a large-scale gene coexpression network (CGN) based on gene expression data available from public repositories. Future research is needed to test these hypotheses.

Taking into account that MeJA has a negative effect on cell growth [9,16,19], another hypothesis to consider is that the cells exposed to MeJA might have remained longer in the stationary phase, keeping a higher potential to produce *t*-R, while the non-exposed controls would enter the lag phase, with less production capacity, in line with the results shown in Figure 1. If that were the case, one would expect that the addition of DIMEB after MeJA would lead to the same production as the reference DIMEB + MeJA together, though perhaps delayed. However, as seen in Figure 5A, the production in cultures first exposed to MeJA level off on the 6th day, achieving a significantly lower production than the reference. Thus, this hypothesis is quite unlikely.

### 4.4. Effect of Darkness on t-R Bioproduction

Growth under darkness is another factor that influences the *t*-R production, as shown in Table 1 and Table S4. The darkness significantly increased the production of *t*-R (concentration and specific production) during the photoperiod regardless of the age of the culture (Table 1). The production of polyphenols is significantly affected in medicinal plants by optimal light conditions [65]. The photoperiod is one of the critical environmental factors to which plants adapt through various physiological modifications that alter the accumulation of secondary metabolites. There are examples of medicinal plants for which the photoperiod enhances the accumulation of certain secondary metabolites. *Basella rubra* callus cultures under the 16:8 h photoperiod produced the highest amount of phenolics compared to those in continuous light or dark conditions [66]. However, there are also many studies that report that continuous light/dark conditions are more effective for stimulating bioactive biosynthesis compared to the photoperiod. Total phenolics and flavonoids were accumulated in continuous dark conditions in the cell cultures of *Lisum usitatissimum.* However, the photoperiod also affects the composition. Fonseca et al., [67] revealed that incubation decreased the content of parthenolide and increased the content of total phenolics in *Tanacetum parthenium,* and the photoperiod had an opposite effect. Tusevski et al., [68] showed a marked difference in the production of phenolic acids, flavonols, flavan-3-ols, and xanthones between the photoperiod and dark conditions in *Hypericum perforatum* cultures. They found that dark-adapted cultures increase the production of flavan-3-ol compared to the photoperiod, while the accumulation of phenolic acids and flavonols is favored in photoperiod conditions. Thus, the specific illuminating regime of darkness that increases *t*-R production in grapevine cell cultures clearly represents a benefit for upscaling in bioreactors, decreasing the cost of production and allowing for the use of steel bioreactors.

### 4.5. Scale-Up to 2 L Bioreactor for t-R Bioproduction

The scaling process from flask to bioreactor can produce a decreased production of secondary metabolites [38]. Thus, the biotechnological method and the bioreactor configuration have a critical role in high throughput production.

The optimal operating conditions of a 2 L laboratory bioreactor in batch for the scale-up production of *t*-R show that the tested conditions in which the highest specific and total production are: high aeration of 1.5 vvm without agitation, a temperature of 24 °C, and 30 g L^−1^ sucrose as a carbon source (Figure 6). Different studies used *Vitis* cell cultures grown in different laboratory bioreactors from 2 L to 14 L for *t*-R production [69]. The conditions used in those studies are 23 to 25 °C for temperature, 0.025 to 0.2 vvm aeration, and 50 to 100 rpm for agitation. Donnez et al. [31] showed the production of *t*-R of 209 mg/L in a MeJA-elicited cell suspension of *Vitis vinifera* cv. Chasselas × Vitis Berlandieri grown in a 2 L stirred bioreactor, which was the highest of the reported studies to date. However, minimal optimization of the operating parameters is reported in those studies.

The present study revealed that aeration of the cell culture in the bioreactor is more important than applying mechanical agitation, even enough to achieve a high *t*-R production. The agitation can have a negative impact on the cell culture due to mechanical damage to elicited cells, as a decrease in cell biomass was detected when the use of agitation was applied (Table 2). The increase in temperature increases the *t*-R production speed. Elicitation at 29 °C showed an exponential increase but stopped after two days, with a maximal *t*-R production of 0.7 mg/mL. An increase in temperature affects enzymatic activity but also the structural stability of the cells, as we observed cell lysis and color change of the media. A low temperature of 19 °C maintains the cell integrity but decreases *t*-R production. Thus, 24 °C is a good compromise between *t*-R production and cellular stress. Regarding the third studied factor, sucrose concentration, 30 mg/mL (the highest concentration tested) produced the highest *t*-R production.

Our results indicate an optimal temperature of 24 °C for the production of *t*-R in elicited grape cell cultures in Biostat B. In order to maintain the metabolic status during the elicitation treatment, the carbon source should not limit *t*-R accumulation, and the results show a minimal carbon requirement of 20 mg/mL sucrose. The tested conditions allowed us to produce up to 2.42 mg/mL. Because this is an experiment in which some operation conditions were tested for their effect on *t*-R bioproduction, further optimization experiments should be performed to find optimal values of these parameters.

## 5. Conclusions

Here, we present the characterization and optimization of the production of *t*-R in grape cell cultures (*Vitis vinifera* cv. Gamay) stimulated with an elicitor mixture of DIMEB and MeJA. We have analyzed a number of factors that may have an impact on *t*-R production in this cell culture in shaken flasks. We may conclude that, in order to maximize the yield, (1) elicitation should be performed on cells in the stationary phase and, as the cell suspension age may have an impact, the production performance should be monitored; (2) in addition to the optimization of elicitor concentration and biomass density, the ratio of DIMEB/biomass has to be optimized as well; (3) the order of addition of the elicitors DIMEB and MeJA matters, which can be related to regulatory mechanisms at the level of gene expression; and (4) if the cell line in use is routinely maintained in the photoperiod, elicitation in darkness may be beneficial. The assays realized in a 2 L commercial stirred tank bioreactor for *t*-R production under DIMEB and MeJA elicitation show that (1) it may work better without mechanical agitation, providing sufficient aeration for an efficient oxygen and mass transfer; (2) a 5 °C increase over the standard growth temperature of 24 °C accelerates the production but at the cost of cell stress and premature collapse, while a 5 °C decrease only affects production yields, without causing collapse; and (3) yields can be enhanced by increasing the sucrose concentration.

On the other hand, the study of the order of addition of elicitors has raised new hypotheses about regulatory mechanisms of stilbene synthesis that will need to be tested in future studies.

## Figures and Tables

**Table 1:** Production values of *t*-R in photoperiod and dark conditions of Gamay grapevine cell suspensions of different ages (Age I: 21 cycles and Age II: 6 cycles) elicited with DIMEB (50 mM) + MeJA (100 μM).

	Age I				Age II			
Cycle	Concentration of *t*-R (mg·mL^−1^)		Specific Production of *t*-R (mg·g biomass^−1^)		Concentration of *t*-R (mg·mL^−1^)		Specific Production of *t*-R (mg·g biomass^−1^)	
	Photoperiod	Darkness	Photoperiod	Darkness	Photoperiod	Darkness	Photoperiod	Darkness
1	3.05 ± 0.09	3.16 ± 0.16	9.75 ± 0.10	10.67 ± 0.32	3.02 ± 0.05	4.24 ± 0.02	9.28 ± 0.09	12.08 ± 0.05
2	3.38 ± 0.03	4.45 ± 0.12	9.70 ± 0.04	13.33 ± 0.14	1.66 ± 0.02	2.90 ± 0.03	5.57 ± 0.04	9.32 ± 0.04
3	3.72 ± 0.08	4.72 ± 0.17	10.99 ± 0.21	14.75 ± 0.18	2.05 ± 0.03	2.67 ± 0.10	6.63 ± 0.08	8.48 ± 0.31
4	3.23 ± 0.03	4.81 ± 0.11	9.69 ± 0.10	14.34 ± 0.12	2.86 ± 0.03	3.65 ± 0.05	8.53 ± 0.04	10.61 ± 0.12
5	4.54 ± 0.14	4.54 ± 0.07	13.16 ± 0.41	14.17 ± 0.08	3.16 ± 0.01	3.46 ± 0.09	9.22 ± 0.03	10.16 ± 0.26
6	3.50 ± 0.09	3.16 ± 0.05	10.12 ± 0.11	9.80 ± 0.05	2.76 ± 0.06	2.95 ± 0.02	8.36 ± 0.14	8.98 ± 0.02
7	4.52 ± 0.03	5.22 ± 0.04	13.10 ± 0.02	15.59 ± 0.08	2.98 ± 0.06	3.12 ± 0.02	8.84 ± 0.06	9.19 ± 0.02
8	3.27 ± 0.03	4.64 ± 0.07	8.94 ± 0.08	13.86 ± 0.07	1.87 ± 0.01	2.76 ± 0.02	5.44 ± 0.01	7.94 ± 0.05
Avg of 8 cycles	3.65 ± 0.58	4.34 ± 0.76	10.68 ± 1.61	13.32 ± 2.02	2.54 ± 0.59	3.22 ± 0.53	7.73 ± 1.60	9.60 ± 1.32

**Table 2:** Effect of aeration and agitation, temperature, and concentration of carbon source on *t*-R production by grapevine cell culture upon elicitation with DIMEB in the Biostat B.

Trans-Resveratrol	Aeration (vvm)/ Agitation Speed (rpm)			Temperature (°C)			Sucrose (g·L^−1^)		
	(0.8/80)	(1.7/80)	(1.7/0)	19	24	29	15	20	30
Concentration (mg·mL^−1^)	1.90	1.60	2.42	1.15	1.60	0.70	1.31	1.65	2.26
Specific production (mg·g biomass^−1^)	5.7	5.6	7.1	3.7	5.6	2.2	4.9	5.3	7.3
Total production (mg)	1425.0	1408.0	1780.9	916.0	1408.0	553.0	1218.3	1336.5	1827.4
Specific productivity ^a^ (mg·g biomass^−1^·day^−1^)	1.43	1.41	1.78	0.9	1.41	1.1	0.97	1.07	1.46

^a^ Calculated using data on day 4 except condition 29 °C, which is calculated using data on day 2.

## Data Availability

No new data were created or analyzed in this study. Data sharing is not applicable to this article.

## Supplementary Materials

The following supporting information can be downloaded at: https://www.mdpi.com/article/10.3390/biom13101529/s1, Figure S1. UV chromatograms (306 nm) obtained by HPLC-U*V*/*v*IS of elicited broths in photoperiod and dark conditions. Figure S2. Fresh weight variation of the elicited cultures maintaining a constant ratio of eliciting agent (0.5 g DIMEB g^−1^ Biomass) for the production of *t*-R at different cell densities. Figure S3. Visual effect of elicitation of grapevine cell suspensions for the production of *t*-R maintaining a constant ratio of eliciting agent (0.5 g DIMEB g^−1^ Biomass) at different cell densities. Table S1. Description of experiment conditions for tR production upon elicitation with DIMEB using Gamay grapevine cell suspension in Biostat B bioreactor with 1.2 L operating volume. Table S2. Average values of concentration and specific production of trans- *t*-R, and analysis of variance of factorial design 3^2^. Table S3. Statistical analysis of darkness effect for *t*-R production in grapevine suspension cells Gamay (paired *t*-test was carried out with the SigmaPlot ver 8.02 software). Table S4. Statistical analysis of age effect for *t*-R production in grapevine suspension cells Gamay (paired *t*-test was carried out with the SigmaPlot ver 8.02 software).
