# Supplementary material for: Factors Affecting the Bioproduction of Resveratrol by Grapevine Cell Cultures under Elicitation
Source: Biomolecules. 2023 Oct 16;13(10):1529. doi: 10.3390/biom13101529 (PMC10605596; doi:10.3390/biom13101529)
Supplement: Supplementary file 1 [file biomolecules-13-01529-s001.zip › biomolecules-2664254-supplementary.pdf]

Supplementary material

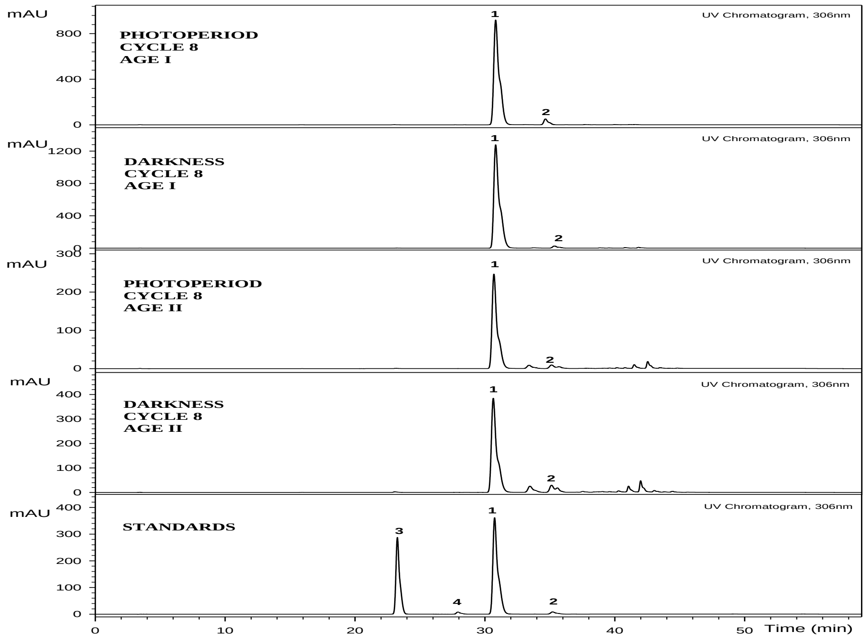

Figure S1. UV chromatograms (306nm) obtained by HPLC-UV/VIS of elicited broths in photoperiod and dark conditions. Peaks: (1) trans-resveratrol, (2) cis-resveratrol, (3) trans-piceid, (4) cis-piceid.

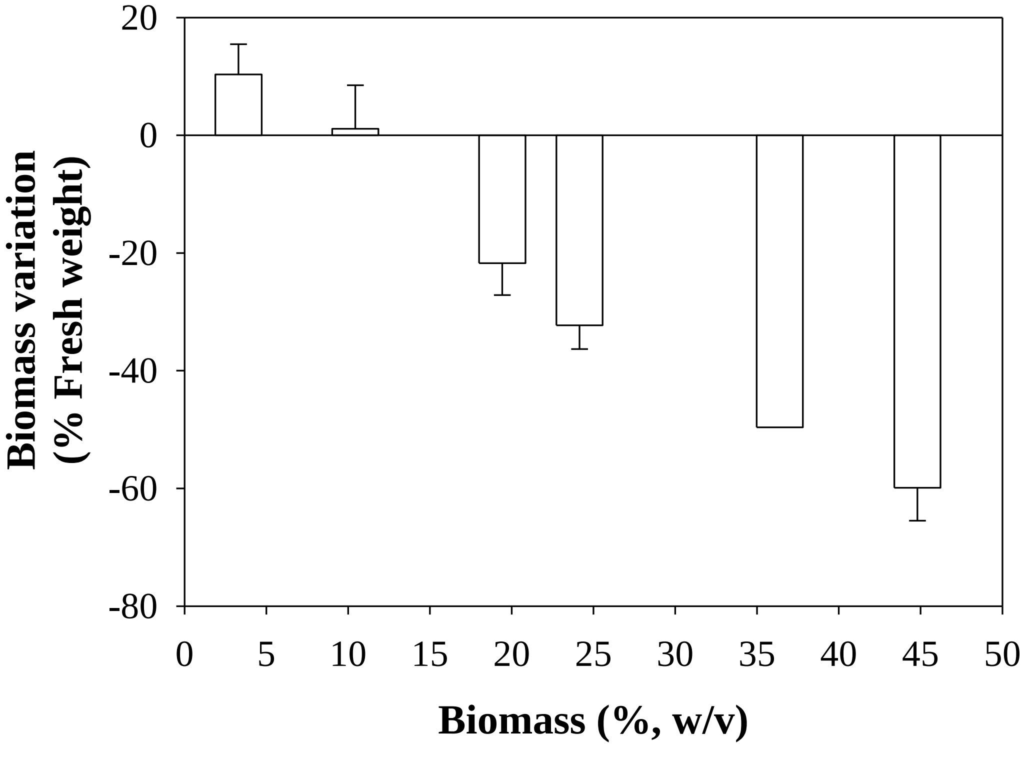

Figure S2. Fresh weight variation of the elicited cultures maintaining a constant ratio of eliciting agent (0.5 g DIMEB. g^{-1} Biomass) for the production of resveratrol at different cell densities.

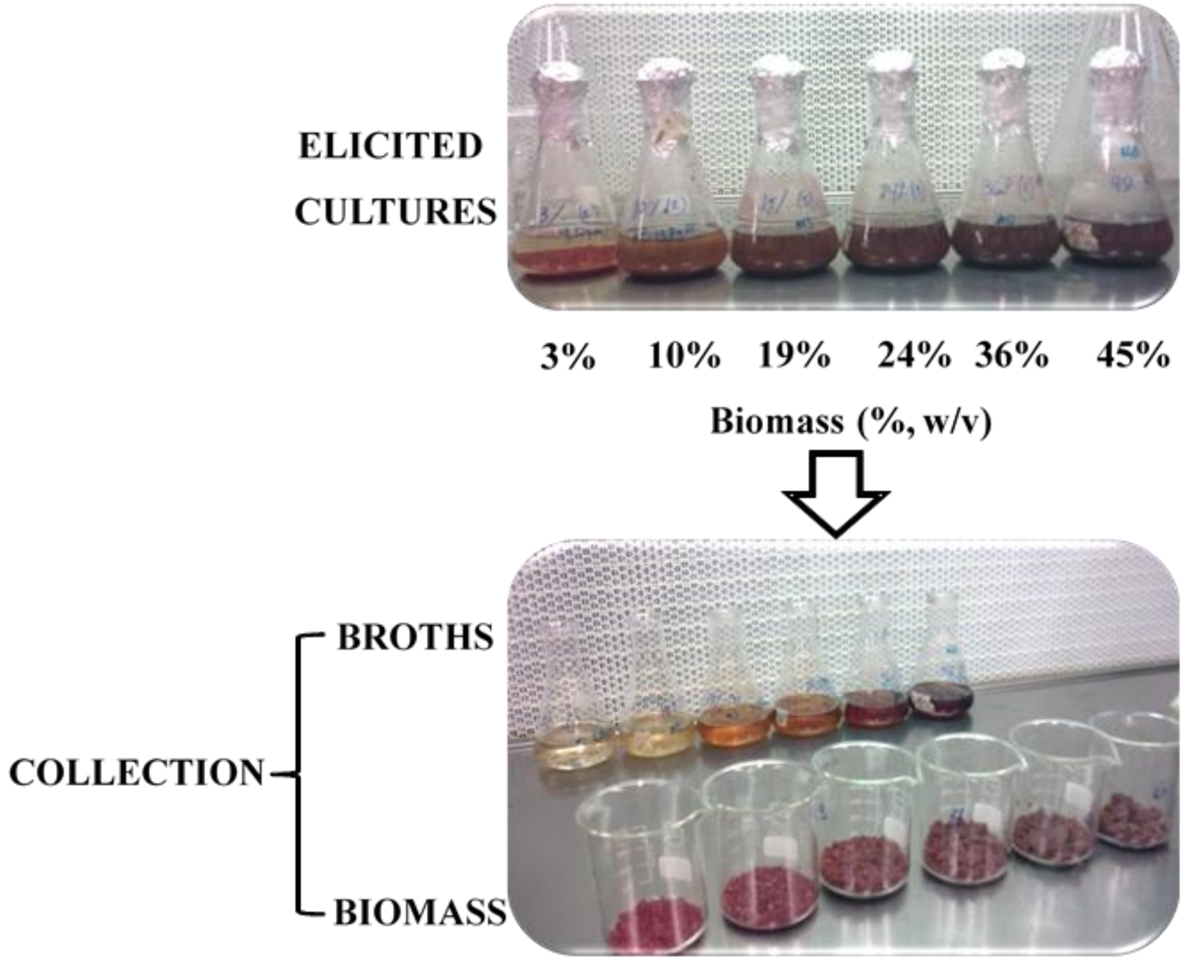

Figure S3. Visual effect of elicitation of grapevine cell suspensions for the production of resveratrol maintaining a constant ratio of eliciting agent (0.5 g DIMEB. g^{-1} Biomass) at different cell densities.

Table S1. Description of experiment conditions for tR production upon elicitation with DIMEB using Gamay grapevine cell suspension in Biostat B bioreactor with 1.2 L operating volume.

	**VARIATION OF PARAMETERS**								
	**Aeration-Agitation**			**Temperature**			**Carbon source**		
**Temperature (ºC)**	**24**	**24**	**24**	**19**	**24**	**29**	24	24	24
**Sucrose ($g.L^{-1}$)**	20	20	20	20	20	20	**15**	**20**	**30**
**Aeration flow rate (vvm)**	**0.8**	**1.7**	**1.7**	1.7	1.7	1.7	1.7	1.7	1.7
**Agitation speed (rpm)**	80	80	0	80	80	80	80	80	80
**Elicitation time (d)**	4	4	4	5	4	1.7	5	5	5
**Photoperiod**	16 h light and 8 h of darkness								
**Biomass (% FW, w/v)**	≈20.5								

Table S2. Average values of concentration and specific production of trans-resveratrol, and analysis of variance of factorial design 3^2.

		Concentration of *t*R (mg.mL^{-1})			Specific Production of *t*R (mg. g FW biomass^{-1})		
		A:DIMEB					
		**(15mM)**	**(35mM)**	**(50mM)**	**(15mM)**	**(35mM)**	**(50mM)**
**B:MeJA**	**(25µM)**	0.57±0.09	2.58±0.23	2.90±0.48	1.9±0.3	9.4±0.9	10.7±1.7
	**(50µM)**	1.78±0.13	3.29±0.43	3.65±0.68	6.7±0.5	12.4±1.5	13.8±2.3
	**(100µM)**	2.08±0.17	3.42±0.14	**4.18±0.14**	8.2±0.8	13.5±0.7	**16.3±0.9**

Analysis of Variance and F-test

Variation Source	gl	SC	MC	F	P		gl	SC	MC	F	P	
**A**	2	21.785	10.892	94.592	<0.001	**	2	311.099	155.549	87.296	<0.001	**
**B**	2	7.010	3.505	30.437	<0.001	**	2	132.803	66.401	37.265	<0.001	**
**AB**	4	0.466	0.117	1.012	0.427	NS	4	5.426	1.356	0.761	0.564	NS
**Residual**	18	2.073	0.115				18	32.073	1.782			
**Total**	26	31.334	1.205				26	481.401	18.515			

Significance codes: >99.9% ‘**’; non significant ‘NS’

Table S3. Statistical Analysis of darkness effect for tR production in grapevine suspension cells Gamay (paired t-test was carried out with the SigmaPlot ver 8.02 software).

	AGE I								AGE II							
	**Concentration (mg.mL^{-1})**				**Specific Production (mg.g biomass^{-1})**				**Concentration (mg.mL^{-1})**				**Specific Production (mg.g biomass^{-1})**			
**Treatment**	**N**	**Mean**	**Std Dev**	**SEM**	**N**	**Mean**	**Std Dev**	**SEM**	**N**	**Mean**	**Std Dev**	**SEM**	**N**	**Mean**	**Std Dev**	**SEM**
Photoperiod	8	3.651	0.577	0.204	8	10.681	1.615	0.571	8	2.545	0.588	0.208	8	7.734	1.604	0.567
Darkness	8	4.338	0.762	0.269	8	13.314	2.024	0.715	8	3.218	0.533	0.188	8	9.595	1.316	0.465
Difference	8	-0.686	0.694	0.245	8	-2.633	1.922	0.680	8	-0.673	0.436	0.154	8	-1.861	1.170	0.414
t	|-2.798|				|-3.873|				|-4.364|				|-4.501|			
df	7				7				7				7			
P value	0.027				0.006				0.003				0.003			
H_0	Rejected				Rejected				Rejected				Rejected			

Critical value of t(0.05; 7)=2.36 (Distribution t table, source: Miller and Miller, 2002). N: number of data. Std Dev: Standard Deviation. SEM: Square Error Media.

Table S4. Statistical Analysis of age effect for tR production in grapevine suspension cells Gamay (paired t-test was carried out with the SigmaPlot ver 8.02 software).

	PHOTOPERIOD								DARKNESS							
	Concentration ($mg.mL^{-1}$)				Specific Production ($mg.g\ biomass^{-1}$)				Concentration ($mg.mL^{-1}$)				Specific Production ($mg.g\ biomass^{-1}$)			
**Treatment**	**N**	**Mean**	**Std Dev**	**SEM**	**N**	**Mean**	**Std Dev**	**SEM**	**N**	**Mean**	**Std Dev**	**SEM**	**N**	**Mean**	**Std Dev**	**SEM**
Age I	8	3.651	0.577	0.204	8	10.681	1.615	0.571	8	4.338	0.762	0.269	8	13.314	2.024	0.715
Age II	8	2.545	0.588	0.208	8	7.734	1.604	0.567	8	3.218	0.532	0.188	8	9.595	1.316	0.465
Difference	8	1.106	0.641	0.227	8	2.947	1.565	0.553	8	1.120	1.087	0.384	8	3.719	2.763	0.977
t	$\vert 4.879 \vert$				$\vert 5.326 \vert$				$\vert 2.915 \vert$				$\vert 3.807 \vert$			
df	7				7				7				7			
P value	0.002				0.001				0.023				0.007			
H_0	Rejected				Rejected				Rejected				Rejected			

Critical value of t(0.05; 7)=2.36 (Distribution t table, source: Miller and Miller, 2002). N: number of data. Std Dev: Standard Deviation. SEM: Square Error Media.
